# Excitatory-inhibitory imbalance in temporal lobe epilepsy: a 5T multimodal MRI biomarker for focus localization and drug resistance stratification

**DOI:** 10.3389/fnagi.2025.1660608

**Published:** 2025-11-13

**Authors:** Zhaodi Huang, He Deng, Ying Xu, Lu Shi, Qingjun Jiang, Ke Xue, Ling Li, Ying Wei, Xiaona Xia, Xiangshui Meng

**Affiliations:** 1 Department of Radiology, Mengchao Hepatobiliary Hospital of Fujian Medical University, Fuzhou, China; 2 Department of Radiology, Qilu Hospital (Qingdao), Cheeloo College of Medicine, Shandong University, Qingdao, China; 3 Department of Radiology, Qilu Hospital of Shandong University, Jinan, China; 4 MR Research Collaboration, Shanghai United Imaging Healthcare Co., Ltd., Shanghai, China; 5 Department of Neurology, Qilu Hospital (Qingdao), Cheeloo College of Medicine, Shandong University, Qingdao, China; 6 Medical Imaging and Engineering Intersection Key Laboratory of Qingdao, Qingdao, China

**Keywords:** excitatory-inhibitory imbalance, temporal lobe epilepsy, GABA, glutamate, MRS, GluCEST

## Abstract

This study aimed to investigate the diagnostic value of combined glutamate chemical exchange saturation transfer (GluCEST) imaging and γ-aminobutyric acid (GABA)-edited proton magnetic resonance spectroscopy (^1^H-MRS) in localizing epileptogenic foci and differentiating drug-resistant epilepsy (DR) from drug-responsive epilepsy (DRES) in temporal lobe epilepsy (TLE) using 5T MRI.

Twenty-four TLE patients (13 left, 11 right) and 25 age-/gender-matched healthy controls (HCs) underwent GluCEST and MEGA-PRESS MRS at 5T MRI. Directional asymmetry indices (DAIglu_H for hippocampus, DAIglu_A for amygdala) and a novel composite biomarker (DAIglu_GABA) integrating GluCEST asymmetry and GABA/Cr ratios were analyzed. Another asymmetry metric was employed to discriminate the left and right TLE groups [DAIglu_H(epi) for hippocampus, DAIglu_A(epi) for amygdala]. Subgroup comparisons (HC vs. DR vs. DRES) and receiver-operating characteristic (ROC) analyses were performed.

**Results:**

GluCEST-derived hippocampal asymmetry [DAIglu_H(epi)] effectively lateralized epileptogenic foci (AUC = 0.86). The DRES patients exhibited elevated DAIglu_H (adjusted *p* < 0.001) and reduced GABA/Cr (adjusted *p* = 0.015) compared to HCs. The DAIglu_GABA index increased in the DRES subgroup compared to HCs (adjusted *p* < 0.001). Moreover, DAIglu_GABA levels were found to be significantly lower in the DR subgroup in comparison to the DRES subgroup (adjusted *p* = 0.009).

**Conclusion:**

Multimodal 5T MRI integrating GluCEST and GABA-MRS provides a clinically feasible tool for lateralizing epileptogenic foci and stratifying drug resistance in TLE. The observed excitatory-inhibitory imbalance dynamics suggest distinct neurometabolic profiles underlying DR and DRES, advancing personalized therapeutic strategies.

## 1 Introduction

Epilepsy is one of the most common and disabling chronic neurologic disorders, affecting approximately 70 million worldwide (Klein et al., 2024). Temporal lobe epilepsy (TLE) is the most common form of focal epilepsy, with about 30%–50% of these patients showing resistance to antiseizure medication, leading to substantial negative health outcomes (George et al., 2024).

For patients with TLE, precise localization of the epileptogenic zone (EZ) is critical, as identification of an magnetic resonance imaging (MRI)-detectable lesion or histopathologically confirmed abnormality confers a 2- to 3-fold greater probability of achieving favorable postoperative outcomes (Davis et al., 2015). However, approximately one-third of patients with TLE exhibit no detectable lesion on conventional MRI (“MRI-negative”) (Gleichgerrcht et al., 2025). Histopathological abnormalities were identified in 87% of those members undergoing resection, indicating undetected epileptogenic lesions and underscoring the limited detection sensitivity of current neuroimaging modalities (Cohen-Gadol et al., 2006). The absence of a lesion complicates the etiological diagnosis of epilepsy and preoperative evaluation for surgical candidates. These findings strongly support the need to develop next-generation imaging tools for mapping epileptic network.

In the healthy adult brain, the balance between excitatory and inhibitory (E/I) signaling is tightly regulated and essential for maintaining network dynamics. γ-aminobutyric acid (GABA) serves as the primary inhibitory neurotransmitter and glutamate (Glu) functions as the primary excitatory neurotransmitter (Van van Hugte et al., 2023). Both human and animal studies indicate that E/I imbalance is a key mechanism in epileptogenesis (Wang et al., 2021; Shimoda et al., 2023). TLE is consistently associated with disrupted glutamatergic and GABAergic circuits, potentially contributing to seizure initiation or maintenance (Ragozzino et al., 2005). Imbalances in these systems may lead to excessive metabolic activation, resulting in excitotoxicity, epileptogenicity, and neuronal death. This process can accelerate seizure spread and expand epileptogenic networks, affecting both seizure-generating and contralateral regions (Xie et al., 2025).

The Mescher–Garwood point-resolved spectroscopy sequence (MEGA-PRESS) enables the detection of GABA distinct from other metabolites (Mullins, 2014). Previous studies using MEGA-PRESS have identified GABA as a potential biomarker for lateralization and monitoring seizure frequency in MRI-negative TLE (Wu S. et al., 2023). In contrast to the distinct singlet resonances of N-acetyl-aspartate (NAA) and creatine (Cr), Glu exhibits triplet or higher-order multiplet resonances, leading to smaller, broader spectral peaks across a wider frequency range. Additionally, Glu’s spectral overlap with glutamine (Gln) hinders accurate interpretation, thereby limiting the clinical utility of magnetic resonance spectroscopy (MRS) techniques for precise Glu measurement.

In recent years, glutamate chemical exchange saturation transfer (GluCEST) has emerged as a powerful tool for localizing epileptogenic foci in temporal lobe epilepsy (TLE), owing to its high sensitivity to metabolic microenvironments and superior spatial resolution (Van Zijl et al., 2021). Compared to conventional ^1^H-MRS, GluCEST offers over 100-fold greater sensitivity for detecting glutamate (Van Zijl et al., 2021). Clinical studies have reported significantly elevated glutamate levels in the epileptogenic hippocampus of TLE patients relative to the contralateral side, consistent with intracranial EEG and pathological findings (Davis et al., 2015).

Previous *in vivo* studies on neurometabolites in human epilepsy have predominantly employed single-modality approaches–either MRS or GluCEST–to assess isolated changes in glutamate or GABA, while neglecting to examine their bidirectional regulatory interactions. It is widely recognized that reduced inhibition, increased excitation, or both contribute to E/I imbalance in epilepsy (Van van Hugte et al., 2023). Prior research using stereo-electroencephalography data has identified pronounced E/I imbalance in the hippocampus and amygdala of patients with drug-resistant epilepsy (DR) (Ooi et al., 2023). GluCEST can localize epileptogenic core regions with Glu level changes, while GABA MRS quantifies the extent of inhibitory neurotransmitter deficiency. Thus, a multimodal imaging strategy combining GluCEST and GABA MRS holds promise for elucidating epileptogenic networks through the lens of E/I balance. We hypothesize that the Glu-to-GABA ratio obtained through non-invasive multimodal MRI techniques may reflect changes in the E/I balance to some extent. This change could potentially indicate the course of epilepsy and may help differentiate between DR and drug-responsive epilepsy (DRES).

Recent advancements in ultra-high-field 7T MRI have further illuminated epileptogenic mechanisms in TLE, demonstrating superior lesion detection and functional mapping. 7T resting-state fMRI has been shown to effectively lateralize seizure-onset zones in TLE cohorts, outperforming 3T in paired comparisons (Lucas et al., 2025). Consensus guidelines from the 7T Epilepsy Task Force emphasize optimized protocols for epilepsy management, reporting higher detection rates for epileptogenic foci (Hangel et al., 2024). Compared to 3T MRI, 5T MRI holds the potential to better localize these lesions and detect previously undiscoverable abnormalities by providing higher resolution, superior contrast (T2*), and increased sensitivity to metabolites using MRS or CEST. However, while 7T offers unparalleled signal-to-noise ratio and contrast for diagnostic accuracy, its clinical adoption is limited by accessibility (few installed systems globally, high costs), safety concerns (elevated specific absorption rate leading to tissue heating risks, contraindications for certain implants), and technical challenges like B1 inhomogeneity (Van Lanen et al., 2021). In contrast, the 5T MRI system, which has been approved for clinical use and supports whole-body imaging, represents a compromise, offering higher resolution and sensitivity while being more feasible for routine clinical deployment. The higher field strength of 5T confers distinct technical advantages for both GABA MRS and GluCEST imaging, including improved spectral resolution and stronger CEST contrast due to increased chemical shift dispersion and longer T_1_ relaxation. These improvements enhance the detection sensitivity for subtle neurotransmitter changes such as GABA, Glx, and glutamate. Therefore, this study aims to (1) utilize GluCEST to predict seizure lateralization in TLE and (2) combine GABA-MRS and GluCEST to investigate the dynamic E/I interactions in TLE patients, while differentiating DR from DRES based on 5T MRI.

## 2 Material and methods

### 2.1 Participants

**FIGURE 1:**
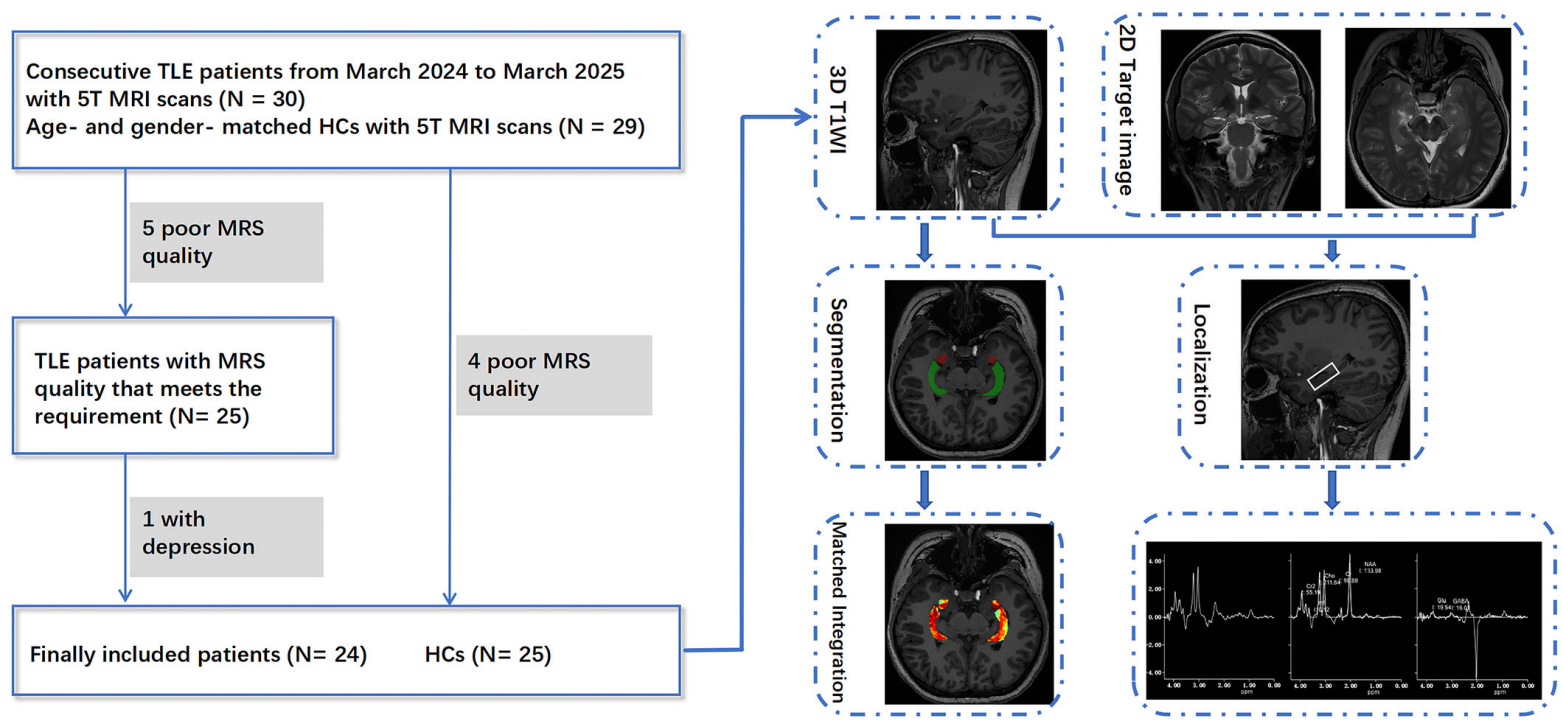
Participant selection and post-processing workflow for TLE patients and healthy controls in this study.

We prospectively enrolled consecutive patients hospitalized with unilateral TLE in the Neurology Department of our hospital from March 2024 to March 2025, based on the following inclusion criteria: (1) The clinical diagnosis of unilateral TLE followed International League Against Epilepsy (ILAE) criteria (Scheffer et al., 2017), and were determined through a comprehensive evaluation involving video-EEG telemetry, seizure semiology, neuropsychological assessment, and neuroimaging conducted by epileptologists (Xie et al., 2025); (2) Underwent a 5T brain MRI scan including three-dimensional T1-weighted imaging (3D T1WI), MRS, with/without GluCEST imaging; (3) Absence of other brain or psychiatric diseases. (4) All patients were right-handed. Exclusion criteria included: (1) Brain parenchymal lesions identified on MRI other than hippocampal sclerosis (HS), such as tumors or vascular malformations; (2) comorbid other mental disorders, such as depression or anxiety; (3) Poor image quality. Inclusion criteria of healthy controls (HCs) were the following: (1) Underwent 5T brain MRI scans including 3D-T1WI, MRS, with/without GluCEST imaging; (2) No personal or family history of psychiatric disorders, and matched to the patient group by age and gender. (3) All controls were right-handed. The exclusion criteria were as follows: (1) brain parenchymal lesions; (2) Poor image quality. In total, 24 patients with unilateral TLE (13 left TLEs and 11 right TLEs) and 25 age- and gender-matched HCs were included in the study, with 19 TLE patients and 23 HCs completing both GluCEST and MRS protocols. The detailed selection process is shown in Figure 1. Patients were categorized as having DR if they continued to experience seizures despite receiving appropriate pharmacological treatment with two or more antiepileptic medications (Cafri et al., 2025).

### 2.2 Magnetic resonance imaging acquisition

All participants underwent MRI scans on a 5T MRI system (uMR Jupiter, United Imaging Healthcare, Shanghai, China) with a 48-channel head coil. Scanning was performed at least 72 h after the last reported seizure. The anatomical imaging protocol included 3D T1WI (repetition-time/echo-time/inversion-time [TR/TE/TI] = 6.8/2.4/620 ms, flip-angle = 5°, field of view = 244 mm × 220 mm, matrix size = 304 mm × 274 mm, voxel size = 0.8 mm^3^ × 0.8 mm^3^ × 0.8 mm^3^, scan-time = 8 min, 10 s), as well as coronal and axial T2-weighted images (T2WI), which facilitated the localization of spectroscopy. The GluCEST sequence was based on a 2D single-shot fast spin echo readout with a square continuous-wave radiofrequency (RF) saturation pulse. GluCEST images were acquired at saturation offset frequencies of ± 3 ppm, ± 3.4 ppm, and ± 2.6 ppm, with the acquisition at ± 3.0 ppm repeated twice. RF saturation was applied with a B1rms of 4 uT and a duration of 1,000 ms. Other parameters were as follows: TR/TE = 6,000/6.5 ms, flip angle = 120°, field of view = 220 mm × 220 mm, matrix size = 128 × 128, number of averages of 1, and in-plane resolution = 1.72 mm^2^ × 1.72 mm^2^, slice thickness = 11 mm. To correct for magnetic field inhomogeneities, B0Map and B1Map were acquired prior to CEST imaging using the same geometric parameters. The acquisition of GluCEST images, B0Map and B1Map were combined in one protocol, with the acquisition time approximately 3 min, 8 s. The GluCEST map was reconstructed online, incorporating B0 and B1 corrections and calculated using the following equation:


$$\text{GluCEST}=\frac{{\text{S}}_{\mathrm{sat}@-3ppm}-{\text{S}}_{\mathrm{sat}@3ppm}}{{\text{S}}_{\mathrm{sat}@-3ppm}}$$

Where, sat@ ± 3 ppm is the image relative to water saturated at ± 3 ppm and corrected for B0 and B1. Single-voxel MRS data were obtained using a prototype MEGA-PRESS sequence. Prior to scanning, B0 field shimming was performed, followed by frequency calibration to ensure field homogeneity. The voxel of interest (VOI) was positioned in the hippocampus ipsilateral to the lesion (15 mm^3^ × 35 mm^3^ × 15 mm^3^), (Figure 1). A refocusing pulse at 1.9 ppm during ON spectra and at 7.5 ppm during OFF spectra were applied. Dynamic frequency calibration was implemented during acquisition to correct for frequency drift. Other parameters were as follows: TR/TE = 3,000/72 ms, flip angle = 90°, bandwidth = 2,000 Hz, number of samples = 2,048; 100 averages for edit ON and 100 averages for edit OFF, scan time = 10 min, 12 s. Scans proceeded only when the full width at half maximum (FWHM) of the unsuppressed water peak was ≤ 14. Dynamic frequency calibration was implemented during acquisition to correct for frequency drift. Only metabolite spectra FWHM ≤ 10 Hz and spectra with a signal-to-noise ratio (SNR) > 3 were included in this study (Wu S. et al., 2023). Spectral quality metrics for GABA and Glu were detailed in Supplementary Table 3.

### 2.3 Magnetic resonance imaging data processing

The hippocampal volume and total intracranial volume (ICV) were automatically segmented by an image analysis tool named uRP, developed by Shanghai United Imaging Intelligence Co. Ltd (Wu J. et al., 2023). To adjust for inter-individual variability in head size, hippocampal volume was normalized to ICV and expressed as Vol_H (hippocampal volume/ICV). Regions of interest (ROIs) in the bilateral hippocampi and amygdala were manually delineated by two radiologists (performed by ZDH and verified by XNX) on matched 3D T1-weighted sequences to obtain the average GluCEST values. Directional asymmetry indices (DAIglu_H for hippocampus, DAIglu_A for amygdala) were calculated evaluate the asymmetry in GluCEST value between regions ipsilateral and contralateral to the seizure onset zone. The formula is: DAIglu =$\frac{{\text{GluCEST}}_{\text{ipsilateral}}-{\text{GluCEST}}_{\text{contralateral}}}{{\text{GluCEST}}_{\text{ipsilateral}}+{\text{GluCEST}}_{\text{contralateral}}}$. An identical formula was applied to the ipsilateral and contralateral whole-hippocampal volumes to obtain the volumetric asymmetry index DAI_vol_H. HCs were matched to the patient cohort according to the left- and right-sided distribution of seizure onset, ensuring equivalent hippocampal laterality ratios between groups (Supplementary Table 1). Another asymmetry metric was employed to discriminate the left and right TLE groups: Difference asymmetry measurements [DAIglu_H(epi) for hippocampus, DAIglu_A(epi) for amygdala] = $\frac{{\text{GluCEST}}_{\text{left}}-{\text{GluCEST}}_{\text{right}}}{{\text{GluCEST}}_{\text{left}}+{\text{GluCEST}}_{\text{right}}}$. MRS postprocessing and spectral quantification were conducted using the uOmnispace.MR MRS application (United Imaging Healthcare, Shanghai, China). Water suppression was performed using the Hankel singular value decomposition method. Removal of noise and apodization were applied with a Hanning window function, and zero-filling was conducted to extend the time-domain signal to 4,096 points, followed by Fourier transformation. Each spectrum was corrected for frequency shifts using the NAA peak as a reference, and underwent polynomial baseline correction and fixed-phase correction based on Cr, Cho, and NAA peaks. The difference-edited spectrum was obtained by subtracting the ON spectrum from the OFF spectrum, and metabolite signals were quantified using a nonlinear least-squares optimization approach for spectral fitting. GABA/Cr and Glx/Cr ratios (where Glx represents the combined glutamate and glutamine signal) were obtained for the ipsilateral hippocampus and their quotient was calculated as the Glx_GABA = $\frac{\mathrm{Glx}/\mathrm{Cr}}{\mathrm{GABA}/\mathrm{Cr}}$. The E/I balance quantification in TLE was quantified using a novel composite biomarker:


$$\mathrm{DAIglu}\,\_\,\mathrm{GABA}={\frac{{\mathrm{DAIglu}}_{H}^{\left(\mathrm{GluCEST}\right)}}{\mathrm{GABA}/{\mathrm{Cr}}^{\left(\mathrm{MRS}\right)}}}_{.}$$

### 2.4 Statistical analysis

Statistical analyses were conducted using SPSS (version 26) and R statistical program (version 4.2.3). The Shapiro–Wilk test was employed to assess the normality of all quantitative data. Categorical data are shown as frequencies (percentages) and continuous data are presented as mean ± standard deviation or median (interquartile range) based on distribution. Group comparisons between patients with TLE and HCs were performed using two-sample *t*-test or Mann–Whitney test for continuous variables, and chi-square test for categorical variables. Group comparisons (HC, DR, and DRES) were conducted using analysis of covariance (ANCOVA) with Vol_H as the covariate for normally distributed variables (Glx and GABA). For non-normally distributed metrics, rank-transformed non-parametric ANCOVA was employed to evaluate between-group differences in DAIglu_H, DAIglu_A, and DAIglu_GABA after adjustment for DAI_vol_H, as well as in Glx_GABA after adjustment for Vol_H. Both dependent variables and covariates were rank-transformed prior to analysis to mitigate non-normality. *Post hoc* pairwise comparisons were adjusted using the Bonferroni method to correct for multiple testing, with adjusted *p*-values reported where applicable. A two-tailed *p*-value < 0.05 was considered statistically significant. Inter-observer variability analyses was conducted to validate the reproducibility and reliability of the DAIglu_GABA index. The diagnostic accuracy of DAIglu_H(epi), DAIglu_A(epi), and their combined model established through logistic regression (DAIglu_HA) in lateralizing epileptogenic foci was assessed using receiver-operating characteristic (ROC) curve analysis (Supplementary Section 3), with the area under the curve (AUC), sensitivity and specificity as the primary metrics. Additionally, the lateralizing value of DAI_vol_H was also assessed. Comparisons between AUCs were conducted using DeLong’s test.

## 3 Results

### 3.1 Comparison of TLEs and HCs

The demographical and clinical information of TLEs and HCs are shown in Table 1 and Supplementary Table 2. Detailed clinical and imaging characteristics of each patient are provided in Supplementary Table 1. No statistically significant differences in age or gender were observed between the two groups. Compared to HC, TLE group exhibited significantly reduced GABA/Cr (*p* = 0.004), and significantly increased DAIglu_H (*p* = 0.008) and DAIglu_GABA (*p* = 0.002). Glx_GABA levels were marginally elevated in the TLE group compared to HC (*p* = 0.056). No significant group differences were observed for Glx/Cr (*p* = 0.387) or DAIglu_A (*p* = 0.277).

**TABLE 1:** Demographic and clinical information.

	TLE (*n* = 24)	HC (*n* = 25)	*P*-value
Age, years	39.8 ± 15.9	35.9 ± 17.5	0.413^a^
Sex, male/female	13/11	14/11	0.897^b^
Glx/Cr	0.231 ± 0.071	0.248 ± 0.063	0.387^a^
GABA/Cr	0.138 ± 0.060	0.186 ± 0.051	0.004^a^
Glx_GABA	1.742 [1.121, 2.938]	1.250 [1.137, 1.512]	0.056^c^
DAIglu_H	0.062 ± 0.073	0.006 ± 0.055	0.008^a^
DAIglu_A	0.041 ± 0.056	0.008 ± 0.118	0.277^a^
DAIglu_GABA	0.639 ± 0.713	0.034 ± 0.350	0.002^a^
DAIglu_H(epi)	0.004 ± 0.096	0.020 ± 0.051	0.523^a^
DAIglu_A(epi)	−0.004 [−0.059, 0.067]	−0.002 [−0.054, 0.035]	0.658^c^
Vol_H	0.234 [0.212, 0.269]	0.243 [0.223, 0.264]	0.509^c^
DAI_H_vol	−0.016 [−0.041, 0.020]	0.011 [−0.012, 0.028]	0.048^c^
Epilepsy duration, month	42 [10, 108]	NA	NA
AEDs, *n*	2 [1, 2.75]	NA	NA
Presence of MTS on MRI, *n*	5 (20.8%)	NA	NA

*^a^*Two-sample *t*-test;

*^b^*Chi-square test;

*^c^*Wilcoxon test. AED, antiepileptic drugs; MTS: mesial temporal sclerosis; NA, not available.

### 3.2 Comparison among HC, DR and DRES groups

TLE patients were divided into the DR group (*n* = 10, 6 males) and the DRES group (*n* = 14, 7 males). The mean age was 37.43 ± 16.04 years for DRES group and 43.20 ± 15.87 years for DR group. No significant differences in age or sex were observed between these subgroups (*p* = 0.392 and *p* = 0.697, respectively). MRI revealed HS in 5 of 10 patients in the DR subgroup, whereas none of the 14 DRES patients exhibited this finding.

#### 3.2.1 Results of GluCEST imaging

**FIGURE 2:**
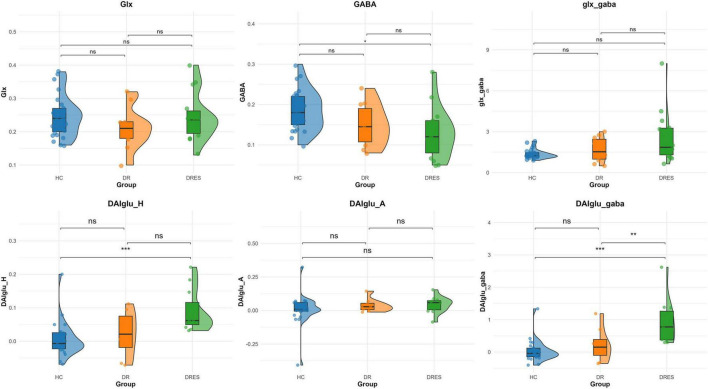
Comparative analysis across HC, DR and DRES groups. Asterisks above group comparisons indicate significant differences based on Bonferroni-adjusted *p*-values: * adjusted *p* < 0.05, ** adjusted *p* < 0.01, *** adjusted *p* < 0.001.

**FIGURE 3:**
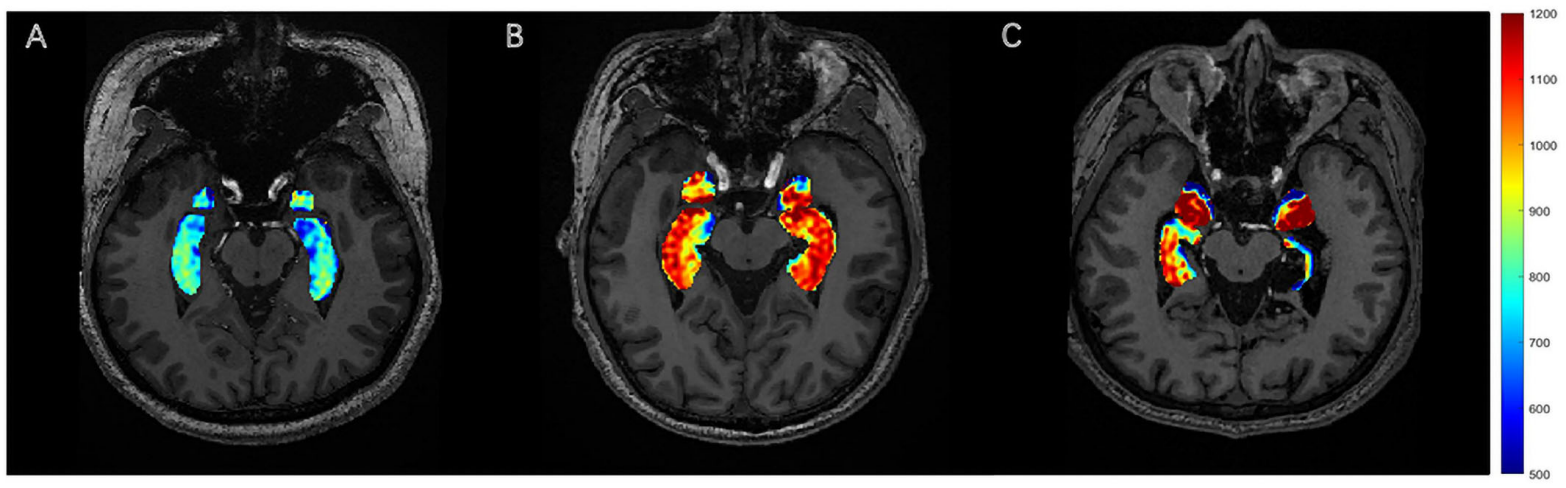
GluCEST signal presentation. **(A)** HC, with no visually discernible differences in GluCEST signal intensity between bilateral hippocampi. **(B)** The DRES patients with left TLE, with a visible increase in the GluCEST signal in the left hippocampus compared to the contralateral side. **(C)** The DR patients with left TLE, with a visible decrease in the GluCEST signal in the left hippocampus compared to the contralateral side.

After adjustment for DAI_vol_H, DAIglu_H was markedly elevated in the DRES subgroup relative to HCs (mean difference = 0.084, 95% CI [0.039 0.130], Cohen’s *d* = 1.454, adjusted *p* < 0.001). A reduction in DAIglu_H within the DR group was observed compared to DRES group, although the difference was not statistically significant (adjusted *p* > 0.05) (Figure 2). No significant differences were found in DAIglu_A among any of the subgroups (all adjusted *p* > 0.05). Notably, 2 patients in the DR group exhibited lower GluCEST values in the ipsilateral hippocampus compared to the contralateral side; these patients, who also presented with visible hippocampal volume atrophy, were among those diagnosed with HS (Figure 3).

#### 3.2.2 Results of MRS

After adjusting for Vol_H, GABA/Cr was significantly lower in the DRES subgroup than in HCs (mean difference = −0.056, 95% CI [−0.095, −0.016], Cohen’s d = −0.984, adjusted *p* = 0.015). Within the DR cohort, GABA/Cr tended to increase and Glx/Cr to decrease relative to the DRES subgroup, although these changes did not reach statistical significance with correction for Vol_H (both adjusted *p* > 0.05) (Figure 2). No significant differences in Glx/Cr or Glx_GABA were detected among any of the subgroups when Vol_H was included as a covariate (all adjusted *p* > 0.05).

#### 3.2.3 Results of combined analysis of GluCEST imaging and MRS

After adjustment for DAI_vol_H, DAIglu_GABA was significantly elevated in the DRES subgroup relative to HCs (mean difference = 0.909, 95% CI [0.458, 1.360], Cohen’s d = 1.876, adjusted *p* < 0.001). Furthermore, when controlling for DAI_vol_H, DAIglu_GABA remained markedly higher in the DRES subgroup than in the DR subgroup (mean difference = −0.723, 95% CI [0.135, 1.311], Cohen’s d = −1.151, adjusted *p* = 0.009) (Figure 2). The intraclass correlation coefficient (ICC) for DAIglu_GABA was 0.890 (95% CI [0.742, 0.956], *p* < 0.001) (see Supplementary Section 4 for details).

### 3.3 DAI for the lateralization of epileptic foci

**FIGURE 4:**
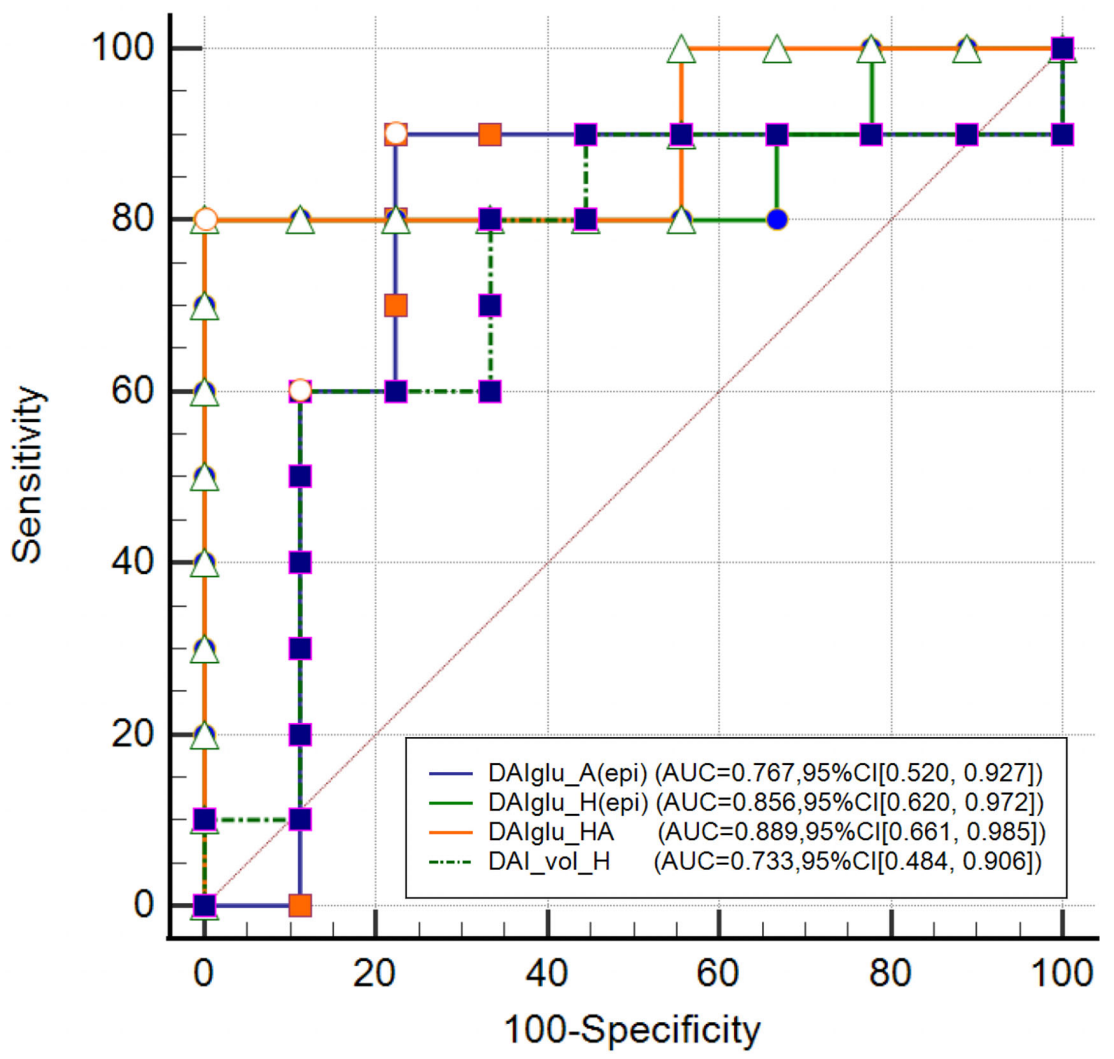
Comparison of DAI_vol_H, DAIglu_A(epi), DAIglu_H(epi) and DAIglu_HA for seizure foci lateralization.

As shown in Figure 4, DAIglu_HA showed the highest AUC of 0.889 (95% CI [0.661, 0.985]), followed by DAIglu_H(epi) (AUC = 0.856, 95% CI [−1.311, −0.135]), DAIglu_A(epi) (AUC = 0.767, 95% CI [0.520, 0.927]), and DAI_vol_H (AUC = 0.733, 95% CI [0.484, 0.906]), although no significant differences were found in the predictive performance. The corresponding sensitivities and specificities were 80% and 100% for DAIglu_H(epi), 90% and 78% for DAIglu_A(epi), 80% and 100% for DAIglu_HA, 69% and 91% for DAI_vol_H. The optimal cutoff value of DAIglu_H(epi) for predicting lateralization was determined to be > 0.0026.

## 4 Discussion

This study is the first to integrate GluCEST and ^1^H-MRS techniques for the lateralization of lesions in TLE and the differentiation between DRES and DR groups using 5T MRI. The results of this study demonstrated that the hippocampal asymmetry index (DAIglu_H) derived from GluCEST could effectively lateralize epileptic foci. Furthermore, this study introduced the DAIglu_gaba index, derived from the combined use of GluCEST and MRS, showed potential for distinguishing between refractory and responsive epilepsy in TLE. In this study, data processing for both examinations utilized relative values instead of absolute values to avoid individual heterogeneity. Given the lengthy acquisition time of MEGA-MRS (approximately 10 min per scan), we proposed a streamlined protocol: GluCEST was first applied for lateralizing epileptic foci, followed by MRS to aid in identifying refractory epilepsy (see Supplementary Section 7 for details). Future longitudinal studies should evaluate this multimodal approach at the point of TLE diagnosis to predict drug refractoriness prospectively, potentially enabling earlier intervention and improved prognostic counseling.

Studies in both humans and animals have shown that Glu levels are as a key manifestation of mitochondrial and metabolic damage in the epileptic network, playing a crucial role in the initiation and maintenance of seizures (Pan et al., 2008; Soukupova et al., 2015). In our study, GluCEST values in the hippocampus or amygdala were elevated on the epileptic side compared to the contralateral side, aiding in the lateralization of the EZ in TLE, which is consistent with the recent findings (Davis et al., 2015; Wang et al., 2023). Microdialysis measurements reveal elevated extracellular glutamate concentrations precisely within the epileptogenic focus (Cavus et al., 2005). Both *in vitro* and *in vivo* investigations have demonstrated impaired glutamate recycling within epileptogenic tissue, a defect that promotes extracellular glutamate accumulation, neuronal hyperexcitability, and excitotoxicity while concurrently depleting intracellular glutamate stores (Petroff et al., 2002a; Cavus et al., 2005). Furthermore, the hippocampal GluCEST asymmetry index was markedly higher in patients with DRES than in HCs. However, in the DR group, the GluCEST values exhibited a paradoxical decrease, as demonstrated in MRS studies of the sclerotic hippocampus (Pimentel Silva et al., 2020). HS is characterized by pronounced granule cell loss and a profound reorganization of excitatory and inhibitory circuits (Petroff et al., 2002b). Reduced hippocampal levels of Glu was also found in chronic animal models of TLE (Alvestad et al., 2008). These findings could explain the lower *in vivo* levels of Glu found in our DR patients compared to DRES patients. The reduced GluCEST values in two DR patients with HS likely reflect glutamatergic synapse loss and impaired synthesis secondary to atrophy, as supported by prior histopathological evidence (Eid et al., 2004). Future analyses incorporating quantitative hippocampal volumetry and histopathological confirmation will be pursued to disentangle HS-specific effects from drug resistance. Our MRS results revealed no significant differences in Glx levels between the TLE and HC groups, consistent with previous studies (Doelken et al., 2008; Leite et al., 2017). This finding may be attributed to disruptions in the Glu-Gln cycle mediated by astrocytes and glutamine synthetase (Ramadan et al., 2013).

GABA is the primary inhibitory neurotransmitter, and abnormalities in the GABAergic system plays a significant role in the pathogenesis of epilepsy (Treiman, 2001; Van van Hugte et al., 2023). In this study, GABA levels were decreased in DRES patients compared to the HCs, whereas an elevation was observed in DR patients, although the difference was not statistically significant. Our findings aligns with a study that reported a reduced GABA/Cr ratio in the bilateral hippocampus of short-duration TLE patients (< 10 years) compared to NCs, with no significant differences observed in long-duration TLE cases (≥ 10 years) (He et al., 2021). Another recent study reported similar findings, showing that patients with TLE had significantly lower GABA levels on the epileptic side compared to the contralateral side and HCs (Wu S. et al., 2023). In patients with juvenile myoclonic epilepsy, thalamic GABA levels were significantly reduced, which was consistent among those receiving novel antiepileptic drugs and sodium valproate treatment (Hattingen et al., 2014). Additionally, the poorer seizure control associated with complex partial seizure disorder and anti-GAD-antibody-positive epilepsy patients appeared to be related to reduced GABA levels (Petroff et al., 2001; Stagg et al., 2010). Furthermore, research on human epileptic brain tissue had provided evidence of reduced GABA-mediated inhibition and decreased GABA concentrations in both cerebrospinal fluid and brain tissue (Treiman, 2001). However, in refractory epilepsy, research utilizing microdialysis had confirmed that GABA levels in the epileptogenic region remained stable (Çavuş et al., 2016), similar to our study. As the disease progresses to chronic stages (long duration), compensatory mechanisms emerge, potentially leading to stabilized or elevated GABA levels (Supplementary Section 6). Histopathological analyses of resected tissue from chronic TLE patients reveal upregulation of glutamate decarboxylase enzymes in surviving GABAergic neurons and even glutamatergic neurons, enhancing GABA synthesis (Houser and Esclapez, 1996). Additionally, axonal sprouting of hippocampal GABAergic interneurons has been observed in mesial TLE models and patients, which may increase overall GABAergic tone as a homeostatic response to recurrent seizures (Alhourani et al., 2020). However, this compensation can be maladaptive; prolonged disease duration is associated with altered chloride homeostasis, including decreased expression of the potassium-chloride cotransporter KCC2 and increased NKCC1, shifting GABA from hyperpolarizing to depolarizing and potentially exacerbating synchrony and epileptogenesis (Palma et al., 2006). These changes are more pronounced in chronically sclerotic hippocampi of adult TLE, where epilepsy-driven plasticity—deafferentation, down-regulation of KCC2, and consequent chloride dysregulation—renders GABAergic signaling depolarizing in subicular pyramidal cells, effectively recapitulating an immature, pro-epileptic state (Cohen et al., 2002). Animal models of chronic epilepsy, such as kainic acid-induced TLE, similarly show elevated GABA in the epileptogenic zone with disease progression, linked to disrupted E/I balance (Hamelin et al., 2021). We hypothesize that in DR progression, elevated GABA exacerbates seizures via GABA_A_ receptor increases, chloride dysregulation, or interneuron hyperactivity promoting oscillations (Cohen et al., 2002; Stamboulian-Platel et al., 2016). Discrepancies across studies, including ours, may arise from confounding factors like disease duration, with early TLE reflecting inhibitory loss and chronic TLE showing paradoxical GABA elevations that fail to restore balance. Future longitudinal studies controlling for duration are needed to clarify these temporal dynamics and their implications for drug resistance.

Based on the E/I balance model of the nervous system, numerous studies had quantified the ratios of GABA to Glu or GABA to Glx as indicators of the E/I balance in specific brain regions (Gu et al., 2019; Bueren et al., 2023). MRS studies have validated E/I imbalance in various neuropsychiatric disorders, supporting its potential as a clinical biomarker (Steel et al., 2020; Pasanta et al., 2023). Our study found no statistically significant differences in the Glx to GABA ratio among the HC, DRES, and DR groups. However, the DAIglu to GABA ratio was able to distinguish between the HC and DRES subgroups, as well as between the DR and DRES subgroups. These findings suggest that Gln signaling may interfere with accurate assessment of E/I balance using Glx, reinforcing the need to separate Glu and Gln signals for more precise interpretation. Our study innovatively developed a novel E/I balance index by integrating Glu levels derived from CEST sequences with GABA levels measured via MRS. Both results were analyzed using relative values to avoid inter-individual heterogeneity and differences in metabolite concentration detection across different sequences. This index effectively captured the E/I imbalance in TLE and yielded results consistent with those from other assessment methods (Xie et al., 2025). The pathophysiology of DR remains multifactorial, with emerging evidence implicating E/I imbalance as a putative mechanism–particularly through pathological shifts in GABA receptor function that may reverse its inhibitory effects (Van van Hugte et al., 2023). While our study focused on drug resistance stratification, we recognize that HS, observed more frequently in the DR subgroup, may contribute to the observed E/I imbalances through mechanisms such as fiber sprouting, GABAergic interneuron alterations, and hippocampal volume loss (Thom, 2014).

This study has several limitations. First, the small cohort size in our study limited the statistical power for subgroup analyses. Second, despite our multimodal combination of GluCEST and MRS providing a novel and practical application for 5T imaging in epilepsy, technical limitations remain. In this study, the GluCEST sequence was limited to a single slice, and the single-voxel MRS could not simultaneously measure Glu and GABA. Moreover, MRS was performed solely on the ipsilateral hippocampus in patients, precluding GABA asymmetry measures and sensitivity analysis. These restrict comprehensive characterization of E/I balance dynamics across distributed epileptic networks. Future studies could address current spatial limitations by adopting advanced 3D or multi-slice imaging protocols. These approaches would enable volumetric coverage and higher-resolution mapping of neurotransmitter distributions across more extensive epileptogenic networks. For GluCEST or MRS techniques in particular, a shift to 3D acquisitions—as demonstrated in recent ultra-high-field studies (Hadar et al., 2021)—could provide multi-regional glutamate asymmetry indices or allow multivoxel quantification of GABA and Glx. Such progress would improve the detection of subtle E/I imbalances within interconnected brain regions, help delineate the spatiotemporal dynamics of E/I dysregulation, and uncover mechanisms underlying seizure propagation. Another limitation was that our TLE diagnoses relied on clinical criteria without invasive monitoring or pathological confirmation, potentially overassuming hippocampal involvement and overlooking bilateral disease where only unilateral seizures were captured. Additionally, potential confounders including interindividual variability in antiepileptic drugs, epilepsy duration, HS and regional gray/white matter volumetric ratios were not systematically controlled, which may have confounded the observed E/I alterations. The concentrations of Glu, Glx and GABA measured by MRI represent the total brain concentrations, including both synaptic and metabolic components (intracellular and extracellular) (Cai et al., 2012; Rideaux, 2021). Since MRI cannot distinguish the sources of neurotransmitters, identifying specific underlying mechanisms remains challenging (Pasanta et al., 2023). Future research requires statistically robust, multi-center cohorts with rigorous control of neurobiological confounders, paired with advanced MRI sequences enabling spatially resolved neurotransmitter quantification across distinct cerebral microdomains, to mechanistically dissect the spatiotemporal dynamics of E/I imbalance in TLE.

## 5 Conclusion

This study establishes the clinical validity of 5T multimodal MRI integrating GluCEST and GABA-MRS for mapping E/I dynamics in TLE. The hippocampal glutamate asymmetry index (DAIglu_H) reliably lateralizes epileptogenic foci, while the composite DAIglu_GABA biomarker discriminates DR from DRES cases, reflecting progressive GABAergic compensation and glutamatergic depletion. Our multimodal sequences hold promise for enhancing the preoperative evaluation of TLE by providing non-invasive information regarding seizure lateralization and drug resistance.

## Data Availability

The raw data supporting the conclusions of this article will be made available by the authors, without undue reservation.
