# Supplementary material for: Excitatory-inhibitory imbalance in temporal lobe epilepsy: a 5T multimodal MRI biomarker for focus localization and drug resistance stratification
Source: Front Aging Neurosci. 2025 Nov 13;17:1660608. doi: 10.3389/fnagi.2025.1660608 (PMC12657467; doi:10.3389/fnagi.2025.1660608)
Supplement: Supplementary file 1 [file Table_1.docx]

**Section 1:**

**Table S1 Clinical and imaging information for TLE patients.**

| Patient No. | Sex | Age (y) | group | Glx/Cr | GABA/Cr | Glx_gaba | DAIglu_H | DAIglu_A | DAIglu_gaba | DAI_vol_H | Vol_H | Epilepsy duration, M | AEDs | MTS on MRI | Localization |
| --- | --- | --- | --- | --- | --- | --- | --- | --- | --- | --- | --- | --- | --- | --- | --- |
| P01 | F | 17 | DRES | 0.34 | 0.16 | 2.125 | 0.221 | 0.069 | 1.383 | -0.023 | 0.228 | 12 | OXC,LEV | No | Right |
| P02 | M | 58 | DRES | 0.24 | 0.16 | 1.500 | 0.061 | -0.007 | 0.383 | -0.009 | 0.272 | 24 | LEV | No | Left |
| P03 | F | 19 | DRES | 0.21 | 0.17 | 1.235 | 0.062 | 0.067 | 0.364 | 0.024 | 0.270 | 10 | LTG,LEV | No | Right |
| P04 | F | 53 | DRES | 0.24 | 0.14 | 1.714 | 0.147 | 0.154 | 1.047 | 0.076 | 0.210 | 6 | LEV | No | Left |
| P05 | F | 62 | DR | 0.15 | 0.24 | 0.625 | 0.069 | 0.049 | 0.286 | 0.020 | 0.223 | 240 | OXC,PMP,VPT | No | Right |
| P06 | M | 27 | DR | 0.23 | 0.09 | 2.556 | / | / | / | 0.031 | 0.286 | 108 | LTG,Mg-VPA,LCM | No | Right |
| P07 | M | 54 | DRES | 0.23 | 0.22 | 1.046 | / | / | / | 0.023 | 0.225 | 360 | LCM | No | Right |
| P08 | F | 61 | DR | 0.23 | 0.13 | 1.769 | / | / | / | -0.255 | 0.153 | 360 | LCM,LEV | Yes | Left |
| P09 | M | 57 | DRES | 0.27 | 0.06 | 4.500 | 0.042 | -0.004 | 0.695 | -0.031 | 0.211 | 120 | CBZ | No | Left |
| P10 | F | 51 | DRES | 0.24 | 0.12 | 2.000 | / | / | / | -0.027 | 0.293 | 24 | LCM | No | Left |
| P11 | M | 33 | DRES | 0.18 | 0.28 | 0.643 | / | / | / | -0.044 | 0.240 | 108 | OXC | No | Left |
| P12 | M | 18 | DR | 0.18 | 0.14 | 1.286 | 0.037 | -0.004 | 0.263 | 0.020 | 0.266 | 10 | LEV,LCM | No | Right |
| P13 | M | 42 | DR | 0.10 | 0.20 | 0.500 | -0.066 | 0.010 | -0.329 | -0.256 | 0.140 | 7 | LCM,LEV | Yes | Left |
| P14 | F | 16 | DRES | 0.19 | 0.05 | 3.800 | 0.058 | 0.015 | 1.156 | -0.032 | 0.214 | 48 | LEV,LTG | No | Left |
| P15 | M | 59 | DR | 0.32 | 0.15 | 2.133 | 0.006 | -0.012 | 0.040 | -0.112 | 0.138 | 48 | LCM,VPT,LEV | Yes | Right |
| P16 | M | 19 | DRES | 0.18 | 0.11 | 1.636 | 0.085 | 0.077 | 0.776 | -0.005 | 0.232 | 120 | LEV | No | Right |
| P17 | F | 54 | DR | 0.22 | 0.08 | 2.750 | 0.095 | 0.068 | 1.186 | -0.058 | 0.163 | 48 | VPT,CBZ,LEV | Yes | Left |
| P18 | F | 44 | DRES | 0.13 | 0.12 | 1.083 | 0.036 | 0.059 | 0.296 | 0.011 | 0.237 | 2 | PMP | No | Right |
| P19 | F | 28 | DR | 0.20 | 0.16 | 1.250 | 0.112 | 0.144 | 0.698 | -0.007 | 0.272 | 48 | VPT,LEV,PB | Yes | Left |
| P20 | M | 45 | DRES | 0.40 | 0.05 | 8.000 | 0.068 | 0.089 | 1.368 | 0.005 | 0.263 | 6 | None | No | Left |
| P21 | M | 34 | DR | 0.18 | 0.20 | 0.900 | -0.071 | 0.025 | -0.355 | -0.027 | 0.241 | 72 | PMP,OXC,LTG | No | Left |
| P22 | M | 32 | DRES | 0.23 | 0.07 | 3.286 | 0.183 | -0.086 | 2.621 | -0.044 | 0.217 | 8 | PMP | No | Left |
| P23 | M | 47 | DR | 0.30 | 0.10 | 3.000 | -0.003 | 0.031 | -0.026 | 0.028 | 0.262 | 14 | LTG,LEV | No | Right |
| P24 | F | 26 | DRES | 0.35 | 0.11 | 3.182 | 0.032 | 0.033 | 0.292 | -0.023 | 0.270 | 36 | OXC | No | Right |

Abbreviations: DRES: drug-responsive epilepsy; DR: drug-resistant epilepsy; AED, antiepileptic drugs; MTS: mesial temporal sclerosis; VPT: valproate; LEV: levetiracetam; LTG: lamotrigine; TPM: topiramate; OXC:  oxcarbazepine; PMP: perampanel; LCM: Lacosamide; Mg-VPA: magnesium valproate; CBZ: carbamazepine; PB: phenobarbital.

**Section 2:**

**Table S2 Clinical information of LTLE/RTLE patients and matched HCs.**

|  | LTLE(n=13) | HC-L(n=14) | *p* | RTLE(n=11) | HC-R(n=11) | *p* |
| --- | --- | --- | --- | --- | --- | --- |
| Age, years | 43.4±13.8 | 40.1±19.3 | 0.633 | 35.6±17.8 | 30.5±14.1 | 0.457 |
| Sex, male/female | 7/6 | 7/7 | 1.0 | 6/5 | 7/4 | 1.0 |

**Section 3: The logistic regression model for DAIglu_HA**

The DAIglu_HA represents a combined model constructed by integrating DAIglu_H and DAIglu_A as independent predictors in a binary logistic regression framework. The model equation is as follows:

$\begin{matrix} \text{log}(\frac{\text{p}}{\text{1-p}})=\text{β}_{0}+\text{β}_{1}\cdot DAI\text{glu}\_H+\text{β}_{2}\cdot DAI\text{glu}\_A \\ \end{matrix}$, where p is the predicted probability of epileptogenic focus lateralization (e.g., left vs. right TLE), β_0_ is the intercept, and β_1_ and β_2_ are the coefficients for DAIglu_H and DAIglu_A, respectively. These predicted probabilities were then used to generate the ROC curve and compute the AUC. The model was fitted using SPSS (version 26), with no multicollinearity issues (variance inflation factors < 2).

**Section 4: Validation of the DAIglu_GABA Composite Biomarker**

To validate the reproducibility and reliability of the DAIglu_GABA index, we conducted inter-observer variability analyses. The DAIglu_GABA index was re-measured by Dr. Deng for the epilepsy group (n=24) using the uRP platform. Inter-observer variability was assessed against original measurements. Subgroup analyses were performed using re-measured data. The intraclass correlation coefficient (ICC) was 0.890 (95% CI [0.742, 0.956], p < 0.001), indicating excellent consistency between observers. The re-measured DAIglu_GABA values were used for subgroup comparisons, which effectively distinguished DR from DRES patients (adjusted p = 0.036) and HCs from DRES patients (adjusted p < 0.001). These findings are consistent with the original conclusions (DR vs. DRES: adjusted p = 0.009; HC vs. DRES: adjusted p < 0.001).

**Section 5: Spectral quality**

Table S3: Spectral Quality Metrics for GABA and Glu

| Group | Metabolite | FWHM (Hz) | SNR |
| --- | --- | --- | --- |
| Epilepsy | GABA | 5.26 ± 2.06 | 4.70 ± 0.90 |
| Epilepsy | Glu | 9.38 ± 1.19 | 6.60 ± 2.82 |
| HC | GABA | 5.81 ± 1.66 | 4.15 ± 1.69 |
| HC | Glu | 9.43 ± 1.33 | 6.18 ± 0.21 |

**Section 6: Correlation between GABA/Cr and disease duration or HS presence**

In epilepsy patients, a weak, non-significant correlation between GABA/Cr and disease duration (Spearman’s ρ = 0.18, p = 0.41) was observed, and patients with ≥60 months disease duration showed higher GABA levels (mean = 0.166) than those with <60 months (mean = 0.124, p > 0.05). Similarly, patients with hippocampal sclerosis (HS) had slightly higher GABA levels than non-HS patients without significance (0.144 vs 0.136, p > 0.05). These non-significant trends, likely limited by small sample size , suggest that chronicity and HS may contribute to elevated GABA/Cr, potentially reflecting compensatory upregulation or chloride transporter dysfunction. Larger cohort studies are needed to validate these preliminary findings.

**Section 7: Clinical Integration of DAIglu_H and DAIglu_GABA Biomarkers**

To translate the DAIglu_H and DAIglu_GABA biomarkers into clinical practice, they can be integrated into existing presurgical assessment protocols for TLE as follows.

DAIglu_H for Epileptogenic Focus Lateralization: DAIglu_H, derived from GluCEST imaging, quantifies hippocampal asymmetry and can serve as a non-invasive adjunct to conventional 3T/7T MRI and video-EEG, particularly in MRI-negative TLE cases. In a typical presurgical workflow, after routine structural MRI and EEG monitoring, patients could undergo 5T multimodal imaging (GluCEST + GABA-MRS, ~15-20 min additional scan time) to compute DAIglu_H. A positive asymmetry threshold (e.g., DAIglu_H > 0.0026, based on our ROC analysis) could corroborate EEG lateralization findings, guiding decisions on invasive monitoring (e.g., stereotactic EEG electrode placement) or direct surgical resection planning. This approach may reduce diagnostic uncertainty and improve postoperative outcomes.

DAIglu_GABA for Drug-Resistance Stratification: The composite DAIglu_GABA biomarker, integrating GluCEST asymmetry and GABA/Cr ratios, distinguishes DR from DRES epilepsy. It could be employed early in the diagnostic pathway, such as after initial AED failure, to stratify patients. For example, elevated DAIglu_GABA values may support continued medical optimization, while lower values could prompt earlier referral to epilepsy surgery centers, aligning with International League Against Epilepsy (ILAE) guidelines for timely intervention in refractory cases. This stratification could optimize patient management by prioritizing surgical evaluation for those unlikely to respond to further AED trials.

Practical Considerations: The implementation of these biomarkers is feasible in epilepsy centers equipped with 5T MRI systems. The additional scan time is minimal, and post-processing is easy to operate via platforms like uRP, as used in this study. To facilitate clinical adoption, standardized thresholds for DAIglu_H and DAIglu_GABA should be validated in prospective multicenter trials. Integration with existing tools, such as PET or functional MRI asymmetry indices, could further enhance diagnostic accuracy. Cost-effectiveness analyses and training for radiologists on 5T protocols will also support widespread adoption.
